# Measles Immunoglobulin G Positivity and Associated Factors Among Suspected Cases in Three Selected Zambian Provinces: A Bayesian Logistic Regression Study

**DOI:** 10.3390/tropicalmed11090248

**Published:** 2026-09-01

**Authors:** Priscilla Nkonde Gardner, Victor Daka, Paul Simusika, Walter Azibadighi, Charles Chileshe, Leah Kamulaza, Davie Simwaba, Roma Chilengi

**Affiliations:** 1 Zambia National Public Health Institute, Lusaka 10101, Zambia; psimusika@yahoo.co.uk (P.S.); chichalesi2@gmail.com (C.C.); leahkamulaza27@gmail.com (L.K.); simwaba74@gmail.com (D.S.); chilengir@yahoo.com (R.C.); 2 Department of Public Health, Copperbelt University, Kitwe 10101, Zambia; dakavictorm@gmail.com; 3 Africa Centres for Disease Control and Prevention, Addis Ababa P.O. Box 3243, Ethiopia; azibadighi@africacdc.org

**Keywords:** measles, vaccination, Bayesian logistic regression, laboratory confirmation, surveillance, Zambia

## Abstract

Measles remains a public-health concern where immunity gaps and incomplete vaccination coverage persist. This study assessed household and geographic factors associated with laboratory-confirmed measles among suspected cases in three Zambian provinces. A prospective cross-sectional study was conducted during measles outbreaks in five selected districts across Luapula, Northern and Northwestern provinces. Suspected cases meeting the IDSR case definition were consecutively enrolled. Blood samples were collected, transported to the UTH Virology Laboratory, and tested using procured IgG test kits. Participant characteristics were summarised descriptively. Bayesian logistic regression estimated associations between laboratory-confirmed (IgG positive) measles and age, sex, vaccination status, household size and previous measles history, with province added in a second model. Results were reported as posterior odds ratios with 95% credible intervals. Among the 172 suspected cases, 45 (26.2%) were laboratory confirmed (IgG positive). Vaccination status was verified using caregiver-provided immunization cards; status was unknown for 110 (64.0%) participants (all of whom tested negative), reflecting missing vaccination documentation. Vaccinated participants had 93% lower adjusted odds of laboratory-confirmed measles than unvaccinated participants (adjusted posterior OR: 0.07, 95% CrI: 0.02–0.18). Adjusted posterior probabilities were consistently lower among vaccinated participants across all three provinces (34% to 49%) than among unvaccinated participants (86% to 92%). Age, sex and household size were not clearly associated with measles positivity. All four participants reporting a previous measles history tested positive, and the unadjusted association was significant using Fisher’s exact test (*p* = 0.004). Vaccination status was most strongly associated with laboratory-confirmed measles, with substantially lower adjusted odds among vaccinated participants. These findings reinforce the importance of measles vaccination in outbreak prevention and control.

## 1. Introduction

Measles remains one of the most contagious vaccine-preventable diseases in the world, and despite the availability of a safe and effective vaccine for over five decades, it continues to cause significant morbidity and mortality among children, particularly in low- and middle-income countries [1]. Although substantial progress has been made globally through routine immunization programs and supplementary immunization activities (SIAs) [2], measles outbreaks continue to occur, particularly in low- and middle-income countries where immunity gaps, suboptimal vaccination coverage, and weaknesses in surveillance systems persist [3]. 

Zambia has struggled to achieve and sustain elimination-level measles–rubella (MR) vaccine coverage. Disruption of routine health services during the COVID-19 pandemic is estimated to have caused over 600,000 Zambian children to miss their first measles-containing vaccine dose, and roughly 500,000 to miss both the first and second doses, between 2020 and 2024 [4]. A subsequent Post-Campaign Coverage Survey conducted across all ten provinces found that, despite the campaign, 11.97% of children aged 9–59 months remained “zero-dose” (unvaccinated against measles and rubella), with the highest prevalence recorded in Central (19.15%) and Western (17.71%) provinces and the lowest in Copperbelt (6.69%) [5]. A post-campaign survey cannot address whether susceptible children seronegative to measles and rubella viruses were vaccinated during the SIA [6].

Methodologically, understanding the individual- and community-level factors that predict measles susceptibility or vaccination status requires an analytical approach suited to binary outcomes (e.g., vaccinated/unvaccinated, case/non-case) while also accommodating the kind of sparse, clustered, and hierarchically structured data typical of provincial-level health surveillance in low-resource settings. Bayesian logistic regression [7] offers such an approach: rather than relying solely on maximum likelihood estimation, it treats model coefficients as random variables with prior probability distributions that are updated with observed data, via Markov Chain Monte Carlo (MCMC) simulation, to yield posterior distributions and credible intervals for each parameter. The Bayesian approach is an adaptive prior that can perform parameter estimation and variable selection well in high-dimensional logistic regression [8].

The Bayesian logistic framework has already been applied productively to closely related questions in African immunisation research. A Bayesian hierarchical (ordinal) logistic regression was used to model the determinants of childhood immunisation coverage across five East African countries using Demographic and Health Survey data, allowing candidate models to be formally compared via leave-one-out cross-validation and coefficients to be interpreted through 95% posterior credible intervals rather than conventional *p*-values [9]. Similarly, a Bayesian multilevel logistic regression was used to identify individual- and community-level predictors of vaccine acceptance in a Ghanaian municipality, combined with spatial mapping to visualise sub-district disparities in uptake [10]. In a related Kenyan study [11], a Bayesian geospatial approach was used to determine subnational inequalities in measles vaccination coverage. Low vaccination coverage and persistent zero-dose clusters are strongly driven not only by supply chain disruptions but also by gaps in vaccine literacy and hesitancy among population subgroups and healthcare personnel, as documented in broader public health evaluations [12]. Understanding these behavioural and knowledge-based determinants is essential for designing effective community engagement strategies to improve immunization uptake in the post-pandemic era.

Zambia’s measles burden is not uniformly distributed. Certain provinces repeatedly emerge as high-risk areas across outbreak surveillance reports, coverage surveys, and serosurveys [5], even as national administrative coverage figures often appear close to elimination targets. This apparent contradiction between reported administrative coverage and actual population immunity and the sub-national clustering of risk provides the rationale for the present study. The study uses Bayesian logistic regression to examine measles epidemiology, immunisation coverage, and associated risk factors in Luapula, Northern and Northwestern provinces of Zambia, with a view to informing more geographically targeted disease control and immunisation strategies.

## 2. Materials and Methods

All children presenting to health facilities in the selected outbreak districts with suspected measles were eligible for enrolment. Suspected measles was defined according to the Zambia Integrated Disease Surveillance and Response (IDSR) case definition: any child presenting with fever, maculopapular rash, and at least one of cough, coryza, or conjunctivitis, or any child in whom a clinician suspected measles infection. This case definition was applied consistently across all participating districts.

### 2.1. Study Population and Inclusion Criteria

All children meeting the inclusion criteria in line with the IDSR case definition who visited the health facilities during the outbreak period were consecutively enrolled in the study.

### 2.2. Data Collection and Specimen Collection

Demographic, clinical, and vaccination history data were collected from enrolled participants using a standardised data collection form. Variables collected included:

(a) Age;
(b) Sex;
(c) Household size;
(d) Vaccination status;
(e) Previous history of measles;
(f) Province and district of residence.

Vaccination status was assessed by asking the caregiver and verifying using the child’s physical immunization card (commonly known as the “under-five card” or “child health card”). All suspected measles cases who presented to health facilities during the outbreak period were enrolled, regardless of whether the caregiver had the immunization card available. Vaccination status was classified as follows:

Vaccinated: Caregiver-reported vaccination and verification using the immunization card documenting at least one dose of measles-containing vaccine.

Unvaccinated: Caregiver-reported lack of vaccination and verification using the immunization card showing no documentation of measles vaccination.

Unknown: No immunization card available at the time of the health facility visit, and vaccination status could not be verified.

Blood samples were collected from each enrolled participant by trained healthcare workers following standard phlebotomy procedures. Samples were handled as follows:

Stored at the recommended temperature (2–8 °C) during field collection.

Transported under appropriate cold chain conditions to the University Teaching Hospital (UTH) Virology Laboratory for testing.

### 2.3. Laboratory Testing

Laboratory confirmation was performed at the University Teaching Hospital (UTH) Virology Laboratory using procured measles-specific IgG test kits (DIA.PRO Measles Virus IgG ELISA) [13]. The DIA.PRO Measles Virus IgG ELISA is a qualitative immunoassay designed for the detection of measles-specific IgG antibodies in human serum or plasma, to determine evidence of immunity following vaccination or previous exposure. The UTH Virology Laboratory serves as Zambia’s national reference laboratory for measles surveillance. Sample Processing and Assay Procedure

Serum samples received for testing were checked for correct identification, sample integrity, and suitability for analysis. Samples were prepared according to the manufacturer’s instructions and diluted 1:101 with the sample diluent.

The DIA.PRO Measles Virus IgG ELISA was performed according to the manufacturer’s procedure. Briefly, the procedure was as follows:

Diluted samples and appropriate controls (blank, negative control, and positive control) were added to the measles virus antigen-coated microplate.

The plate was incubated for 60 min at 37 °C, then washed.

Enzyme conjugate was added, and the plate was incubated for a further 60 min at 37 °C.

After washing, the TMB/H_2_O_2_ substrate was added, and the plate was incubated for 20 min at room temperature, protected from strong light.

The reaction was stopped, and optical density (OD) was measured at 450 nm, with 620–630 nm as the reference wavelength.

Calculation and Interpretation

For the qualitative assay, the cut-off was calculated as follows:

Cut-off = Mean Negative Control OD + 0.250

The negative control OD values were NC1 = 0.075 and NC2 = 0.072, giving a mean negative control of 0.0735. The assay cut-off was therefore calculated as follows:

Cut-off = 0.0735 + 0.250 = 0.3235

The sample/cut-off (S/Co) value was calculated for each sample:

S/Co = Sample OD ÷ Cut-off

According to the manufacturer’s interpretation criteria,

S/Co < 1.0: Negative.

S/Co ≥ 1.0: Positive.

Samples with an S/Co < 1.0 were interpreted as negative, while samples with an S/Co ≥ 1.0 were interpreted as positive.

Interpretation of IgG Results: In the context of suspected measles cases investigated during outbreaks, IgG positivity can indicate the following:

Acute or recent measles infection (IgG appears within 1–2 weeks after rash onset).

Previous measles infection or vaccination (pre-existing immunity).

Secondary vaccine failure in vaccinated individuals.

Unlike IgM testing, which is the WHO-preferred method for acute measles diagnosis, IgG testing alone cannot definitively distinguish between acute infection and pre-existing immunity from past infection or vaccination [12]. This limitation is particularly relevant in settings with previous measles circulation or high vaccination coverage.

Quality Control

Internal quality control was assessed using the blank, kit negative control, and kit positive control. The assay acceptance criteria specified by the manufacturer were as follows:

QC Parameter → Acceptance Criterion

Blank < 0.100 OD at 450 nm.

Negative Control < 0.150 OD at 450 nm.

Positive Control ≥ 0.750 OD at 450 nm.

Mean CV% < 30%.

All QC results met the specified acceptance criteria before sample results were interpreted and reported. Quality assurance was maintained through the following:

Adherence to the manufacturer’s instructions for IgG test kits.

Routine internal quality controls at the UTH Virology Laboratory.

Participation in external quality assessment programs through the WHO Global Measles and Rubella Laboratory Network.

Standardised training of laboratory personnel.

### 2.4. Sample Size

A complete enumeration of 172 suspected measles cases were included in the analytical dataset, of whom 45 tested positive for measles. Records with indeterminate or undocumented laboratory results were excluded from the primary outcome analysis. Participants with unknown vaccination status were excluded from the primary regression analysis so that the estimated vaccination effect represented a direct comparison between participants classified as vaccinated and unvaccinated. The final number included in each regression model was reported because complete information was required for all variables entered in the model.

### 2.5. Outcome Variable

The primary outcome was laboratory-confirmed measles, defined using the recorded laboratory result. The outcome was coded as follows:

$$Y_{i}=\left\{\begin{array}{cc} 1, & \mathrm{laboratory-positive}\;\mathrm{measles}\;\mathrm{result}\\ 0, & \mathrm{laboratory-negative}\;\mathrm{measles}\;\mathrm{result} \end{array}\right.$$

Participants with positive laboratory results were therefore classified as confirmed measles cases, whereas those with negative results formed the comparison group.

### 2.6. Explanatory Variables

The following explanatory variables were considered based on their epidemiological relevance and availability in the surveillance dataset: age, sex, vaccination status, household size, reported previous history of measles and province. Age was analysed as a continuous variable. To improve model estimation and facilitate comparison with other continuous predictors, age was standardised using the following:

$$\mathrm{Age}\;\mathrm{z-score}=\frac{\mathrm{individual}\;\mathrm{age}-\mathrm{mean}\;\mathrm{age}}{\mathrm{standard}\;\mathrm{deviation}\;\mathrm{of}\;\mathrm{age}}$$

The resulting odds ratio was interpreted as the change in the odds of laboratory-confirmed measles associated with a one-standard-deviation increase in age. 

Sex was treated as a categorical variable, with female as the reference category and male as the comparison category. Vaccination status was categorised as unvaccinated and vaccinated. Unvaccinated participants were used as the reference category. Participants whose vaccination status was recorded as unknown were not included in the primary regression analysis. The coefficient for vaccinated participants therefore represented the odds of laboratory-confirmed measles among vaccinated participants relative to unvaccinated participants, after adjustment for the other variables in the model.

Household size was analysed as a continuous variable and standardised in the same way as age:

$$\mathrm{Household-size}\;\mathrm{z-score}=\frac{\mathrm{household}\;\mathrm{size}-\mathrm{mean}\;\mathrm{household}\;\mathrm{size}}{\mathrm{standard}\;\mathrm{deviation}\;\mathrm{of}\;\mathrm{household}\;\mathrm{size}}$$

Its odds ratio was interpreted per one-standard-deviation increase in household size.

Reported previous measles history was classified as no and yes. Participants reporting no previous measles history formed the reference category. Province was treated as a categorical variable with Luapula Province as the reference category. 

### 2.7. Descriptive and Statistical Analysis

Participant characteristics were summarised using frequencies and percentages for categorical variables. Continuous variables were summarised using the mean and standard deviation when approximately normally distributed, or the median and interquartile range when the distribution was skewed. Differences in continuous variables between participants with IgG-positive and IgG-negative laboratory results were assessed using the Mann–Whitney U test because the variables did not meet the assumptions required for an independent-samples t-test. Categorical variables were compared using Pearson’s chi-square test where expected cell counts were adequate, while Fisher’s exact test was used for sparse comparisons or when expected cell counts were small.

Bayesian logistic regression was used to estimate associations between participant characteristics and laboratory-confirmed measles. The primary model included age, sex, vaccination status, household size and previous measles history. A second model additionally adjusted for province to assess whether geographic differences influenced the magnitude or direction of the estimated associations. Data management, statistical analysis and visualisation were performed in Python version 3.9.7. 

Bayesian logistic regression analysis

Bayesian logistic regression was used to estimate associations between participant characteristics and laboratory-confirmed measles. For participant i, the laboratory result $Y_{i}$ was modelled as a Bernoulli outcome [13], such that $Y_{i}∼\mathrm{Bernoulli}\left(p_{i}\right)$, where $p_{i}$ represents the probability of testing positive for measles. The probability was related to the explanatory variables through the logit link:

$$\log\left(\frac{p_{i}}{1-p_{i}}\right)=\beta_{0}+\overset{K}{\underset{k=1}{\sum }}\beta_{k}X_{ki},$$

where $\beta_{0}$ is the intercept, $X_{ki}$ represents the value of predictor k for participant i, and $\beta_{k}$ is the corresponding regression coefficient. The primary model included standardised age, sex, vaccination status, standardised household size and previous measles history. A second model additionally adjusted for province to assess whether geographic differences influenced the estimated associations. 

Weakly informative normal priors were assigned to the regression coefficients, $\beta_{k}∼\mathrm{Normal}\left(0,0.7\right)$. These priors permitted associations in either direction while regularising extreme estimates arising from the small number of IgG-positive cases and sparse categories, particularly previous measles history, for which all four exposed participants tested positive. Posterior distributions were estimated using Markov chain Monte Carlo sampling with the No-U-Turn Sampler implemented through Bambi and PyMC [13]. Regression coefficients were exponentiated to obtain posterior odds ratios:

$${\mathrm{OR}}_{k}=\exp\left(\beta_{k}\right).$$

Results were summarized using posterior odds ratios, 95% credible intervals and posterior directional probabilities, expressed as $P\left(\mathrm{OR}>1\mathrm{data}\right)\;$for estimated positive associations or $P\left(\mathrm{OR}<1\mathrm{data}\right)\;$for estimated inverse associations. A 95% credible interval was interpreted as containing 95% of the posterior probability for the parameter, conditional on the observed data, model, and prior distributions. Model convergence was assessed using trace plots, rank-normalized $\hat{R}$, bulk and tail effective sample sizes and the number of divergent transitions. Trace plots for the province-adjusted sensitivity model are presented in Figure S1.

Convergence was considered satisfactory when all $\hat{R}$ values were below 1.01, effective sample sizes were adequate, and no divergent transitions were observed. Posterior predictive checks were also conducted to assess whether the fitted models adequately reproduced the observed distribution of laboratory-positive and laboratory-negative cases.

### 2.8. Ethical Considerations

Ethical approval for the study was obtained from the ERES Converge IRB (Ref: 2026-May-041; Approval Date: 1 June 2026) and National Health Research Authority of Zambia (Ref: NHRA-3664/01/06/2026) shown in Appendix A and Appendix B. The study was conducted in accordance with the Declaration of Helsinki and national guidelines for research involving human participants. Informed consent was obtained from parents or legal guardians of all enrolled children prior to study procedures.

## 3. Results

Of the 172 suspected measles cases included in the analysis, 45 (26.2%) were laboratory confirmed (IgG positive). Participant characteristics stratified by laboratory result are presented in Table 1. Age and household size were comparable between IgG-positive and IgG-negative participants (*p* = 0.518 and *p* = 0.0.214, respectively). Laboratory positivity differed substantially by vaccination status, with a greater proportion of positive cases among unvaccinated participants (*p* < 0.001). Vaccination status was unknown for 110 (64.0%) participants, all of whom tested IgG negative, reflecting missing vaccination documentation (no immunization card available for verification). All four participants reporting a previous history of measles tested positive, and previous measles history was associated with laboratory positivity in the unadjusted analysis (Fisher’s exact *p* = 0.004).

Factors associated with laboratory-confirmed measles in the fully adjusted Bayesian logistic regression model.

Factors associated with laboratory-confirmed measles were assessed using the fully adjusted Bayesian logistic regression model, which included age, sex, vaccination status, household size, previous measles history and province (Table 2). Vaccination status showed the strongest and most clearly supported association with laboratory-confirmed measles. After adjustment for the other covariates, vaccinated participants had substantially lower odds of testing positive compared with unvaccinated participants (posterior OR 0.07, 95% CrI 0.02–0.18). The estimated associations for age, sex, household size and previous measles history were less precise, with 95% credible intervals including the null value. The estimated association for previous measles history was highly imprecise (posterior OR: 1.24, 95% CrI: 0.35–4.54), reflecting the very small number of participants (n = 4) with a reported previous history of measles. Although all four of these participants tested IgG positive, the wide credible interval indicates considerable uncertainty in the magnitude of this association.

**Figure 1:**
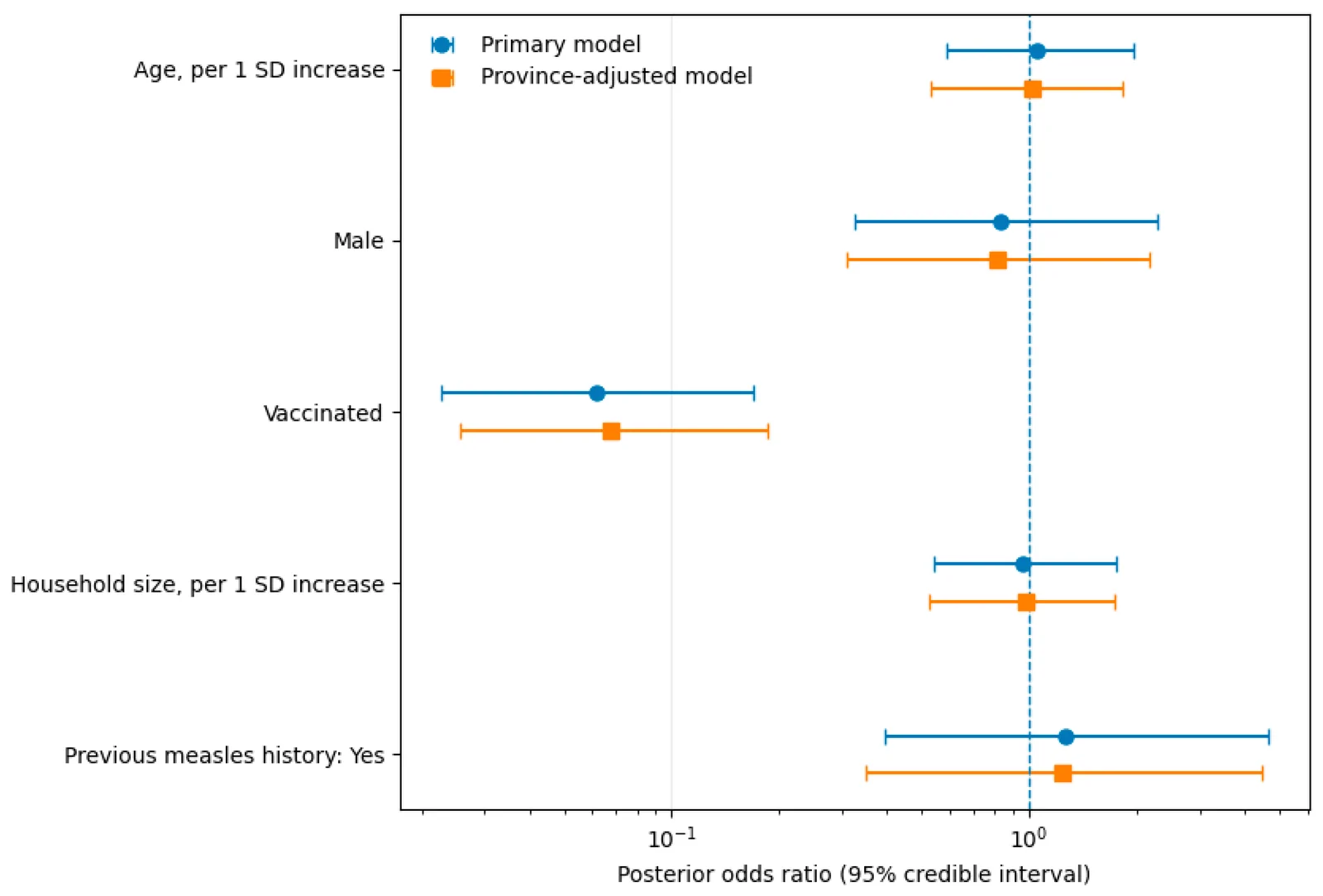
Posterior odds ratios from the primary and province-adjusted models.

In the primary Bayesian logistic regression model, vaccination was strongly associated with reduced odds of laboratory-confirmed measles. The posterior estimates for age, sex, household size and previous measles history were imprecise, with 95% credible intervals spanning the null value. In the province-adjusted Bayesian logistic regression model (Figure 1), vaccination status remained strongly associated with laboratory-confirmed measles. The magnitude and direction of the vaccination effect were materially unchanged after adjustment for province. In addition, the posterior credible intervals for the province coefficients did not include the null value, indicating meaningful differences in measles positivity between the included provinces and the reference province.

**Figure 2:**
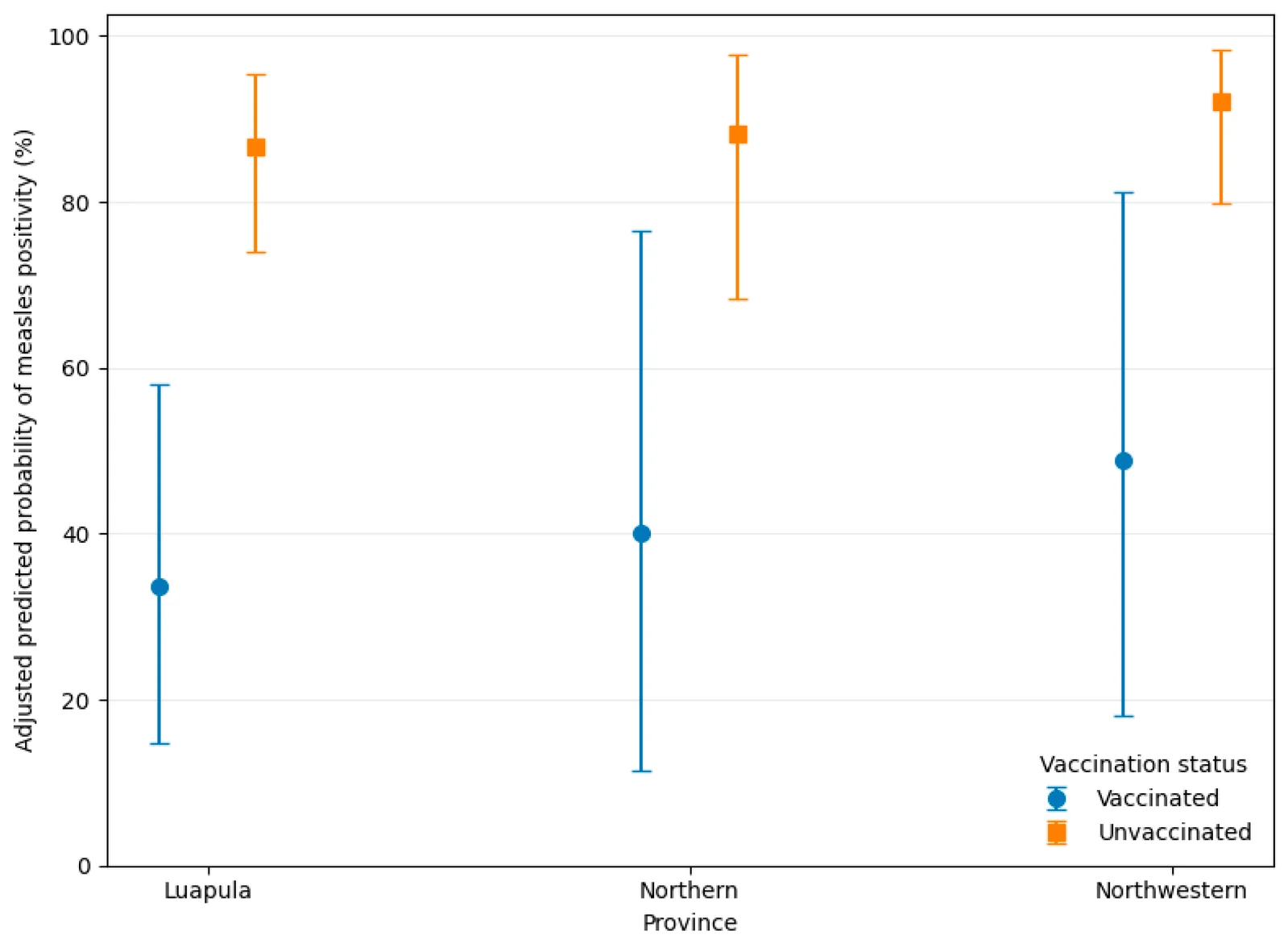
Adjusted posterior probability of laboratory-confirmed measles by vaccination status and province.

The adjusted posterior probability of laboratory-confirmed measles was substantially higher among unvaccinated than vaccinated participants across all provinces (Figure 2). Among unvaccinated participants, the estimated probability of positivity ranged from approximately 86% in Luapula to 92% in Northwestern Province, compared with approximately 34% to 49% among vaccinated participants. Although predicted positivity appeared somewhat higher in Northwestern Province, the overlapping credible intervals indicated considerable uncertainty in the provincial differences.

## 4. Discussion

This study examined factors associated with laboratory-confirmed measles among suspected cases reported from Luapula, Northern and Northwestern provinces. The principal finding was that vaccination status was strongly associated with measles positivity. Vaccinated participants had substantially lower adjusted odds and lower predicted probabilities of laboratory-confirmed measles than unvaccinated participants. This association remained evident after adjustment for age, sex, household size, previous measles history and province, suggesting that the observed protective association of vaccination was not explained by the measured demographic, household or geographic characteristics.

The adjusted probability analysis further illustrated the importance of vaccination status. Across all three provinces, unvaccinated participants had a considerably higher predicted probability of testing positive than vaccinated participants. Although predicted positivity appeared to vary across provinces, the credible intervals for the provincial estimates overlapped, particularly among vaccinated participants. Therefore, the evidence for differences between provinces was less conclusive than the evidence for differences by vaccination status. Provincial variation may nevertheless reflect differences in vaccination coverage, population movement, outbreak intensity, access to health services, case detection and timeliness of specimen collection. These contextual factors were not fully captured in the dataset and should be considered when interpreting the geographic findings.

The strong inverse association between vaccination and laboratory-confirmed measles is consistent with the biological and public-health role of measles vaccination in reducing susceptibility to infection. However, some vaccinated participants still tested positive. This finding should not necessarily be interpreted as evidence of vaccine failure because the collected data did not provide sufficient information on the number of vaccine doses received, age at vaccination, documentation of vaccination, or time since vaccination. The presence of IgG-positive cases among vaccinated individuals may reflect primary vaccine failure, waning immunity over time, or incomplete dosing schedules rather than true vaccine ineffectiveness. Without serological confirmation of immune status or detailed vaccination records including dates and number of doses received, it is not possible to distinguish between these explanations.

Similar challenges in verifying true protective immunity versus self-reported status have been extensively highlighted in institutional surveillance studies [14], demonstrating that reliance on incomplete immunization records often misclassifies actual susceptibility.

Future analyses should distinguish between zero, one and two documented doses where this information is available.

Age was not clearly associated with measles positivity after adjustment for the other covariates. The posterior estimate was imprecise and its credible interval included the null value. This suggests that the available data did not provide strong evidence that the odds of laboratory-confirmed measles changed with increasing age. Nevertheless, the absence of clear evidence should not be interpreted as proof that age has no epidemiological importance. The relatively small number of confirmed cases, the treatment of age as a linear continuous variable and possible clustering of cases within particular age groups may have limited the ability to detect an age-related pattern. A larger study could examine age using epidemiologically meaningful categories or assess whether its association with positivity is nonlinear.

Similarly, there was no clear evidence of an independent association between sex and laboratory-confirmed measles. The adjusted estimates for males and females were uncertain and compatible with little or no difference. This may indicate that exposure and susceptibility were broadly similar by sex in the study population. However, differences in care-seeking behaviour, surveillance reporting or exposure patterns could still exist and may require a larger sample to evaluate adequately.

Household size was also not clearly associated with measles positivity in the adjusted analysis. Although measles is highly transmissible and household crowding could plausibly increase exposure, household size alone may not accurately represent contact intensity or crowding. Variables such as the number of rooms, sleeping arrangements, presence of school-aged children, contact with a confirmed case and population density may provide more direct measures of transmission opportunity. The absence of a clear association may also reflect limited statistical power and incomplete measurement of household-level exposure.

A particularly notable descriptive finding was that all four participants who reported a previous history of measles tested positive. Fisher’s exact test indicated evidence of an unadjusted association. However, the Bayesian adjusted estimate was highly imprecise and its 95% credible interval included one. This difference arises because the unadjusted analysis considered only the two-way association between previous measles history and laboratory outcome, whereas the regression model simultaneously adjusted for other variables and accounted for uncertainty arising from the very small, exposed group. The pattern also created complete separation because no laboratory-negative participant reported a previous measles history. Although the Bayesian prior produced a finite estimate, the small number of participants meant that the independent association could not be estimated precisely. 

The estimates from the primary and province-adjusted models were generally similar. Adjustment for province did not materially alter the association between vaccination status and measles positivity. This stability supports the robustness of the principal finding within the limitations of the available data. However, the province-adjusted model should not be interpreted as fully accounting for all geographic differences, because only three provinces were represented and unmeasured variation may have existed between districts, facilities and communities.

The use of Bayesian logistic regression was an important strength of the analysis. The dataset contained only 45 laboratory-positive cases and included sparse categories, especially previous measles history. Under these conditions, conventional maximum-likelihood logistic regression can produce unstable or infinite estimates. Weakly informative priors regularised extreme coefficients while allowing the uncertainty associated with limited data to be reflected in the posterior distributions. Reporting posterior odds ratios, credible intervals and directional probabilities also provided a more complete description of the strength and uncertainty of the observed associations than relying solely on dichotomous significance testing.

A notable limitation of this study is the high proportion (64.0%) of participants with unknown vaccination status, all of whom tested IgG negative for measles. This reflects the reality that vaccination documentation is often unavailable during outbreak investigations as caregivers may not have the immunization card at the time of the health facility visit. Several potential biases may arise from this. Caregivers who bring immunization cards may represent a more health-seeking population with higher vaccination coverage, potentially contributing to the observed association between record completeness and negative test results. During outbreak periods, facilities may be overwhelmed, and staff may not have the capacity to request cards from all caregivers. The observation that all 110 participants with unknown vaccination status tested IgG negative means that the “unvaccinated” group consisted exclusively of individuals with documented unvaccinated status. If individuals with unknown vaccination status were disproportionately unvaccinated but misclassified, our effect estimates might overstate the protective effect of vaccination. However, the strong and consistent association between documented vaccination and reduced odds of IgG positivity aligns with the well-established biological efficacy of measles-containing vaccines and is unlikely to be entirely attributable to this source of bias.

An important methodological consideration is the use of IgG rather than IgM testing for laboratory confirmation. While IgM is the WHO-preferred marker for acute measles diagnosis, IgG testing provides valuable information about immune status in suspected cases. However, IgG positivity cannot definitively distinguish between acute infection and pre-existing immunity from past infection or vaccination [14]. This limitation has several implications for our analysis.

First, some IgG-positive cases in our dataset may represent individuals with pre-existing immunity who developed a febrile rash illness from another aetiology (e.g., rubella, parvovirus B19, dengue, or drug reactions) but were investigated as suspected measles. If such cases were disproportionately represented among vaccinated individuals, the observed protective effect of vaccination could be underestimated. Second, the presence of IgG-positive cases among vaccinated individuals does not necessarily indicate vaccine failure; it may simply reflect the persistence of vaccine-induced IgG antibodies in individuals who were investigated for non-measles febrile rash illnesses. Third, the inability to distinguish acute from past infection means that the “previous measles history” variable (self-reported) cannot be independently verified through laboratory testing. The finding that all four participants reporting previous measles history tested IgG positive is therefore not surprising and may reflect either true previous infection or recall bias combined with pre-existing immunity.

Despite these limitations, the strong inverse association between documented vaccination and IgG positivity is consistent with the well-established role of vaccination in maintaining protective antibody levels. However, future studies should prioritize IgM testing or paired acute-convalescent IgG testing to confirm acute measles cases and distinguish them from individuals with pre-existing immunity. The World Health Organization recommends IgM detection as the standard for acute measles surveillance, and strengthening laboratory capacity for IgM testing in Zambia would enhance diagnostic specificity.

The finding that all four participants reporting previous measles history tested IgG positive should be interpreted with caution given the statistical imprecision resulting from the small number of cases. The wide credible interval for this estimate (OR: 1.24, 95% CrI: 0.35–4.54) reflects substantial uncertainty, and the association observed in the unadjusted Fisher’s exact test (*p* = 0.004) does not account for potential confounding factors. Previous measles infection is known to confer immunity, but the validation of self-reported history is challenging, particularly in outbreak investigation settings where recall may be incomplete or inaccurate. The Bayesian framework with weakly informative priors prevented infinite estimates, but the resulting imprecision underscores the need for larger studies with more complete history documentation to precisely estimate this association.

The absence of dose-specific vaccination data (MCV1 versus MCV2) is a notable limitation of this study. Two doses of measles-containing vaccine are recommended to achieve optimal protection, and immunity levels differ substantially between single-dose and two-dose recipients. Our inability to distinguish between these groups may have attenuated the observed protective effect and contributed to the presence of IgG-positive cases among vaccinated individuals. Future surveillance systems should routinely collect information on the number of vaccine doses received, dates of vaccination, and age at vaccination to enable more nuanced analyses of vaccine effectiveness and to identify individuals at risk of incomplete immunization.

Implications for Geographically Targeted Strategies

Our primary aim was to generate evidence to inform more geographically targeted disease control and immunization strategies in Zambia. The strong protective association between vaccination and IgG positivity across all three provinces reinforces the centrality of vaccination as the cornerstone of measles control. However, the provincial-level comparisons in our analysis should be interpreted with caution due to the limited sample size and wide credible intervals. While Northwestern Province appeared to have higher predicted positivity among unvaccinated participants, the overlapping credible intervals mean that the evidence for meaningful provincial differences was inconclusive. This uncertainty highlights a key limitation: the current dataset does not provide sufficient statistical power to precisely estimate provincial-level differences in measles positivity after accounting for individual-level factors.

To fully achieve the aim of informing geographically targeted strategies, larger, more comprehensive datasets with complete vaccination histories and consistent case investigation across all provinces would be required. Future studies should prioritize standardised data collection procedures, including verification of vaccination status through immunization cards or electronic registries, and routine collection of additional covariates such as socioeconomic status, travel history, and healthcare access. Enhanced surveillance systems that collect these variables consistently across all provinces would enable more precise identification of high-risk geographic areas and subpopulations, facilitating targeted interventions. Nevertheless, the present study provides proof of concept for the application of Bayesian methods to Zambian measles surveillance data and demonstrates how such approaches can yield robust estimates even with sparse data, provided that key variables such as vaccination status are adequately recorded.

Study Positioning as a Model for Future Work

This study demonstrates the potential of Bayesian logistic regression as an analytical framework for primary data collection in outbreak settings. The ability to incorporate weakly informative priors, estimate posterior probabilities, and derive credible intervals rather than relying solely on *p*-values makes this approach particularly suited to the sparse, clustered, and hierarchically structured data typical of provincial-level health surveillance in Zambia and similar settings. The application of Bayesian methods to measles surveillance data can serve as a model for future analyses involving larger datasets to assess multiple dimensions of immunization program quality, including coverage completeness, timeliness of vaccination, and geographic equity. By leveraging Bayesian approaches, national immunization programs can generate more robust evidence from existing surveillance systems and outbreak investigations, even when data are incomplete, and use this evidence to identify priority areas for intervention and strengthen health systems.

Recommendations for Enhanced Surveillance Data Collection

To strengthen measles surveillance and immunization program effectiveness in Zambia, we recommend the collection of additional data elements including the following:

Vaccination history details: Number of MCV doses received, dates of administration, and age at vaccination to enable dose-specific vaccine effectiveness analyses and identification of under-vaccinated populations.

Vaccination verification method: Whether status was verified through immunization cards, facility records, or caregiver recall, to distinguish between true unvaccinated status and potential recall or documentation bias.

Socioeconomic indicators: Household wealth, maternal education, and occupation to identify equity gaps in vaccination coverage.

Healthcare access measures: Distance to nearest health facility, usual source of care, and barriers to vaccination to inform supply-side interventions.

Geographic coordinates: GPS coordinates of case residence to enable spatial analysis and identification of transmission hotspots.

Specimen collection timing: Date of rash onset and date of specimen collection to assess the sensitivity of laboratory detection.

Clinical outcome: Hospitalization, complication, and mortality data to assess disease severity and health system impact.

Standardised data collection across all provinces, integrated with electronic immunization registries and routine health information systems, would enable more comprehensive analyses, facilitate real-time outbreak detection, and support evidence-based decision-making for immunization programs.

### Study Limitations

This study has several limitations that should be considered when interpreting the findings:

The use of IgG testing rather than IgM testing means we could not definitively distinguish between acute measles infection and pre-existing immunity from past infection or vaccination.

The high proportion of participants with unknown vaccination status (64.0%) reflects missing vaccination documentation (no immunization card available for verification), which may have introduced bias if card availability was associated with vaccination status or healthcare-seeking behaviour.

The small sample size (n = 172) and the sparse data for certain predictors (e.g., previous measles history, n = 4) resulted in wide credible intervals and imprecise estimates for some associations.

We lacked dose-specific vaccination data and could not distinguish between single-dose and two-dose recipients.

We did not collect data on socioeconomic status, healthcare access, or travel history, which may confound the observed associations.

The study was conducted during outbreak periods in five selected districts, and findings may not be generalizable to non-outbreak settings or other provinces.

## 5. Conclusions

This study found that vaccination status was the strongest factor associated with IgG-positive measles among suspected cases in three Zambian provinces. Vaccinated participants had substantially lower adjusted odds of IgG positivity compared with unvaccinated participants, reinforcing the critical importance of measles vaccination in outbreak prevention and control. The high proportion of participants with unknown vaccination status highlights the need for improved vaccination documentation and record-keeping in outbreak settings. Future surveillance should prioritize IgM testing for acute case confirmation, collect dose-specific vaccination data, and strengthen systems for verifying vaccination status. Bayesian logistic regression proved to be a valuable analytical framework for handling sparse data and estimating associations with appropriate uncertainty, and should be considered for similar analyses in low-resource settings.

## Figures and Tables

**Table 1:** Participant characteristics.

Characteristic	Overall	Positive	Negative	*p*-Value
Total	172	45	127	
Age, years, median (IQR)	6.0 (4.0–8.0)	6.0 (4.0–9.0)	6.0 (4.0–8.0)	0.518 ^†^
Household size, median (IQR)	5.0 (4.0–6.0)	5.0 (5.0–6.0)	5.0 (4.0–6.0)	0.214 ^†^
Province				0.677 ^‡^
Luapula	105 (61.0%)	25 (55.6%)	80 (63.0%)	
Northwestern	54 (31.4%)	16 (35.6%)	38 (29.9%)	
Northern	13 (7.6%)	4 (8.9%)	9 (7.1%)	
Sex				0.766 ^‡^
Female	95 (55.2%)	24 (53.3%)	71 (55.9%)	
Male	77 (44.8%)	21 (46.7%)	56 (44.1%)	
Age group				0.910 ^‡^
<1 year	0 (0.0%)	0 (0.0%)	0 (0.0%)	
1–4 years	48 (27.9%)	12 (26.7%)	36 (28.3%)	
5–9 years	104 (60.5%)	27 (60.0%)	77 (60.6%)	
10–14 years	20 (11.6%)	6 (13.3%)	14 (11.0%)	
≥15 years	0 (0.0%)	0 (0.0%)	0 (0.0%)	
Vaccination status				<0.001 ^‡^
Vaccinated	17 (9.9%)	0 (0.0%)	17 (13.4%)	
Unvaccinated	45 (26.2%)	45 (100.0%)	0 (0.0%)	
Unknown	110 (64.0%)	0 (0.0%)	110 (86.6%)	
Previous history of measles				0.004 ^§^
Yes	4 (2.3%)	4 (8.9%)	0 (0.0%)	
No	168 (97.7%)	41 (91.1%)	127 (100.0%)	

Values are presented as n (%) unless otherwise indicated. ^†^ Mann–Whitney U test; ^‡^ Pearson’s chi-square test; ^§^ Fisher’s exact test. Percentages were calculated within laboratory-result groups.

**Table 2:** Factors associated with laboratory-confirmed measles.

Predictor	Adjusted Posterior OR (95% CrI)	Probability of Increased Odds (%)	Probability of Reduced Odds (%)
Age, per 1 SD increase	1.02 (0.55–1.89)	53.2	46.8
Sex			
Female	1.00 (REF)	REF	REF
Male	0.81 (0.31–2.17)	34.3	65.7
Vaccination Status			
Unvaccinated Vaccinated	1.00 (REF)	REF	REF
	0.07 (0.02–0.18)	0	100
Household size, per 1 SD increase	0.98 (0.53–1.78)	47.4	52.6
Previous measles history			
No	1.00 (REF)	REF	REF
Yes	1.24 (0.35–4.54)	63.2	36.8
Province			
Luapula	1.00 (REF)	REF	REF
Northern	1.30 (0.38–4.36)	66	34
Northwestern	1.97 (0.65–6.28)	87.5	12.5

**Figure A1:**
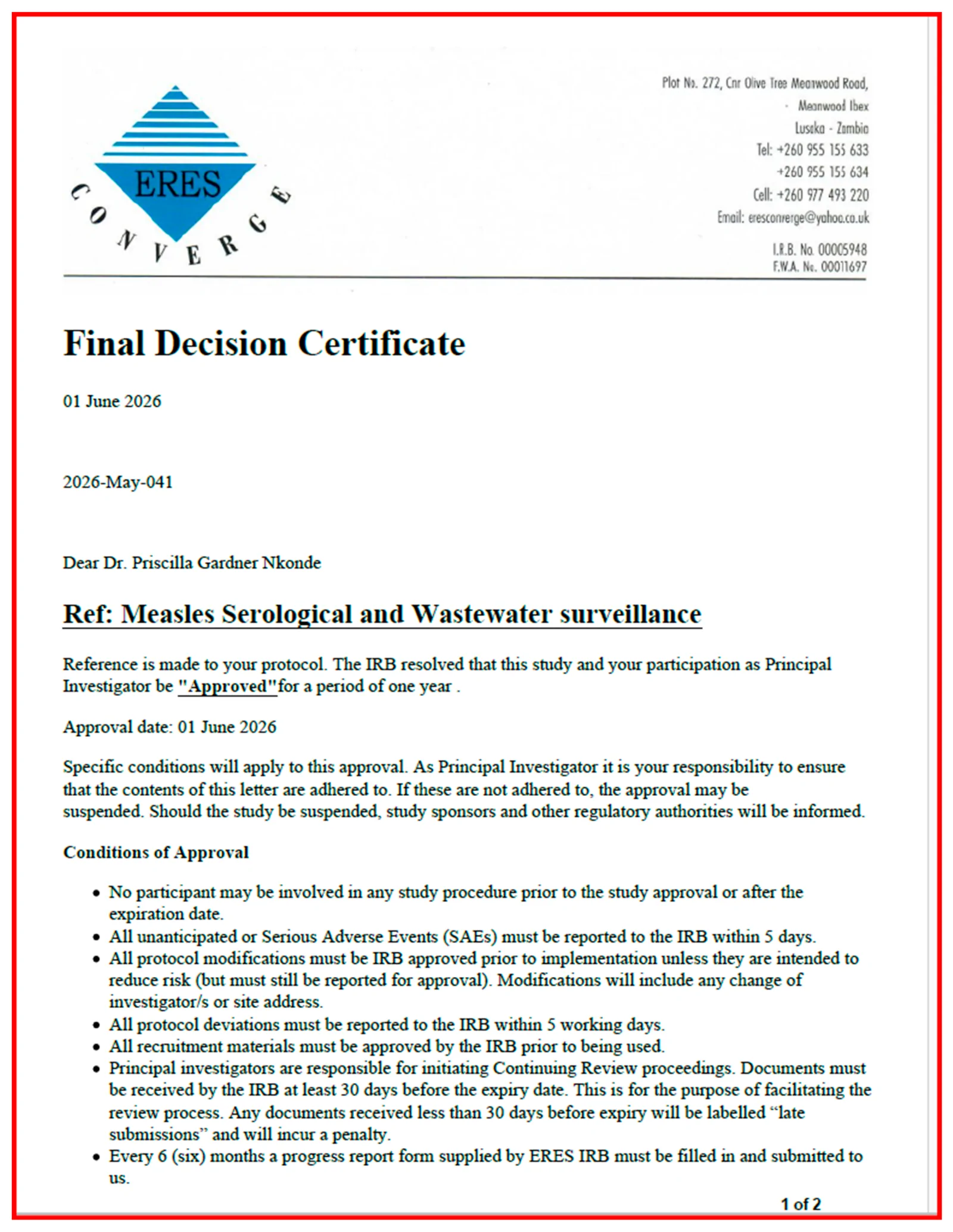

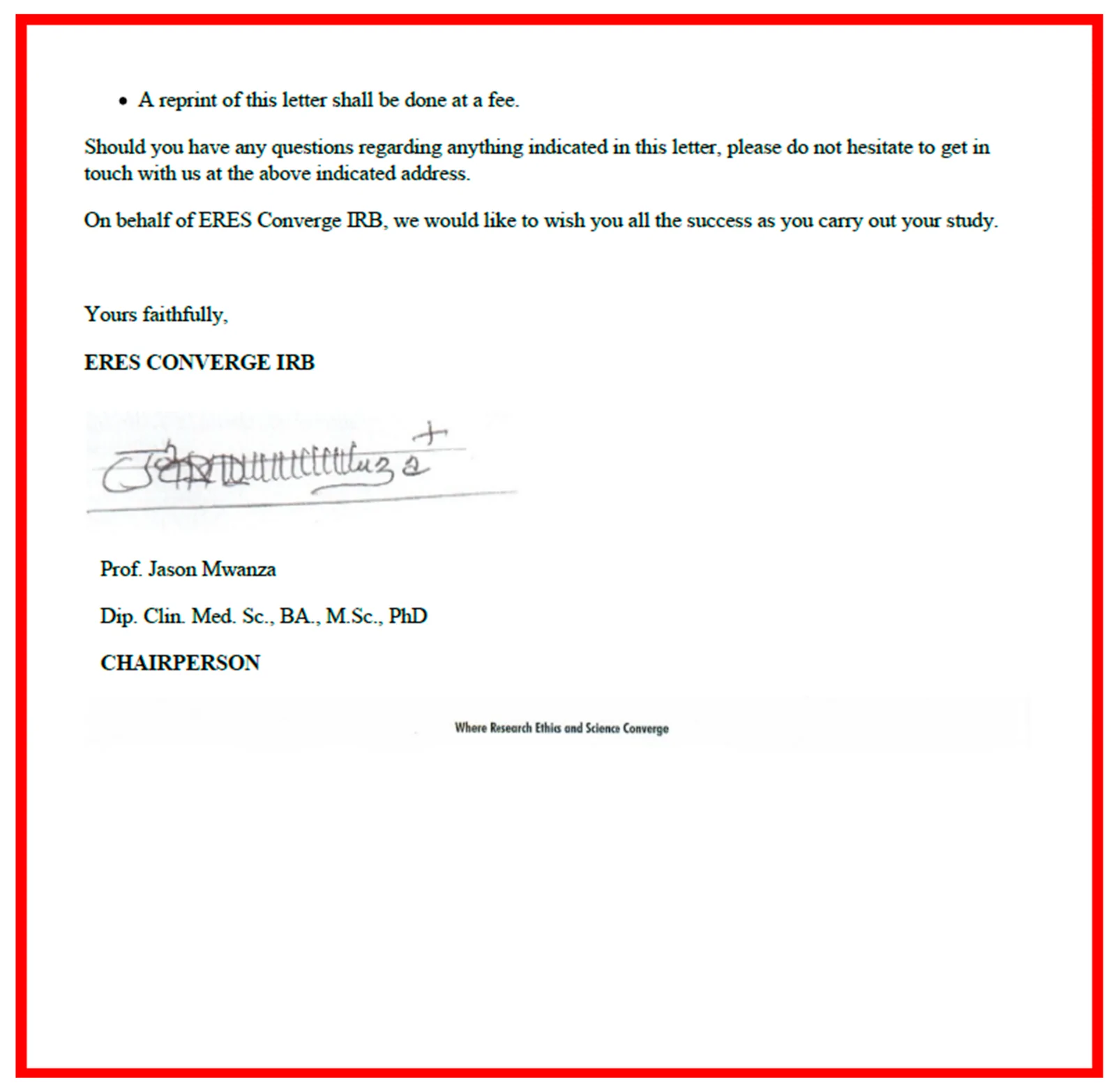
Ethical Approval.

**Figure A2:**
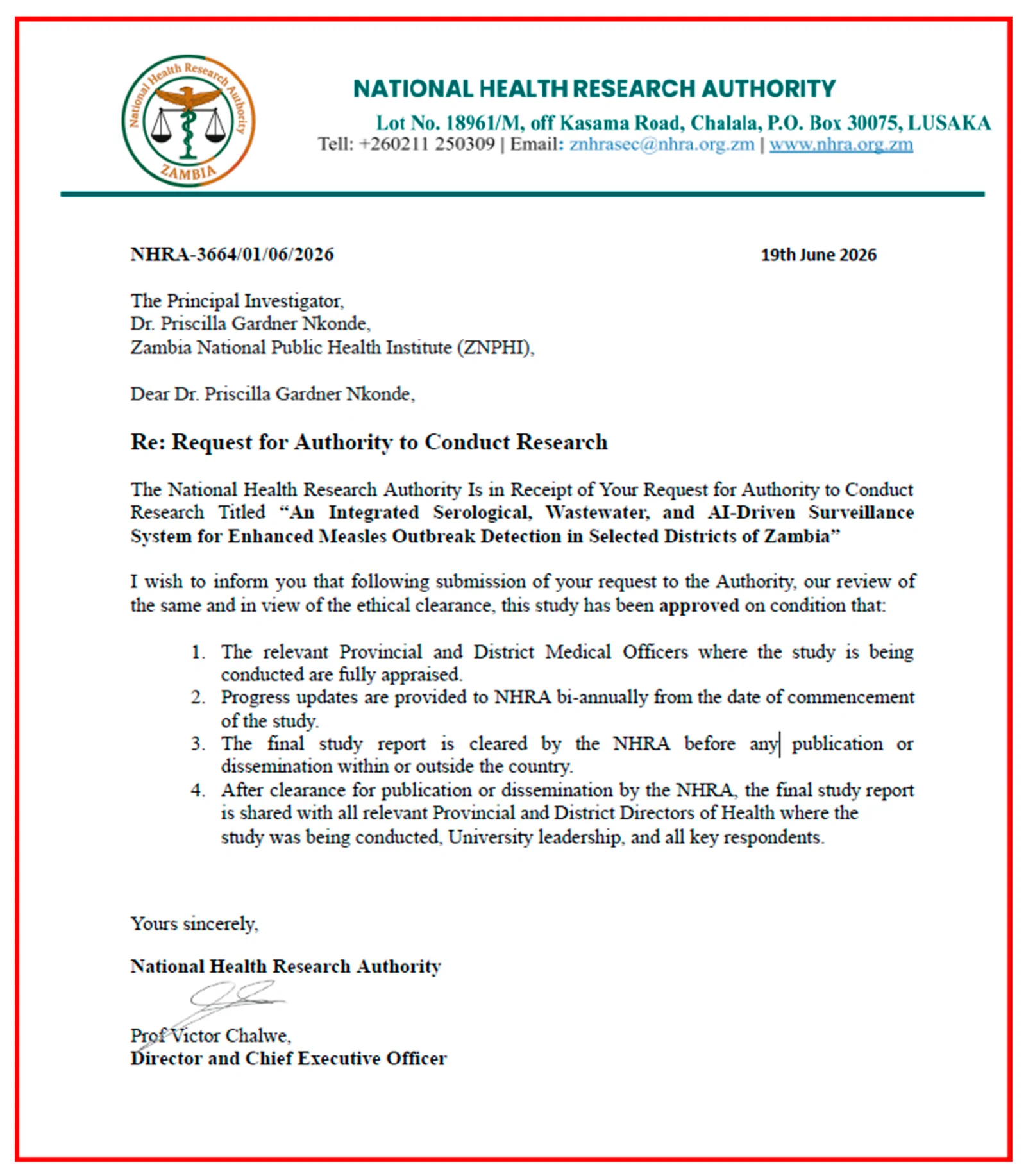
NHRA Approval—National Health Research Authority Clearance (NHRA).

## Data Availability

The datasets generated and/or analysed during the current study are available from the corresponding author upon reasonable request.

## Supplementary Materials

The following supporting information can be downloaded at: https://www.mdpi.com/article/10.3390/tropicalmed11090248/s1, Figure S1: Trace plots for province-adjusted sensitivity model.
