# Supplementary material for: Measles Immunoglobulin G Positivity and Associated Factors Among Suspected Cases in Three Selected Zambian Provinces: A Bayesian Logistic Regression Study
Source: Trop Med Infect Dis. 2026 Sep 1;11(9):248. doi: 10.3390/tropicalmed11090248 (PMC13611125; doi:10.3390/tropicalmed11090248)

Figure S1: Trace plots for province-adjusted sensitivity model

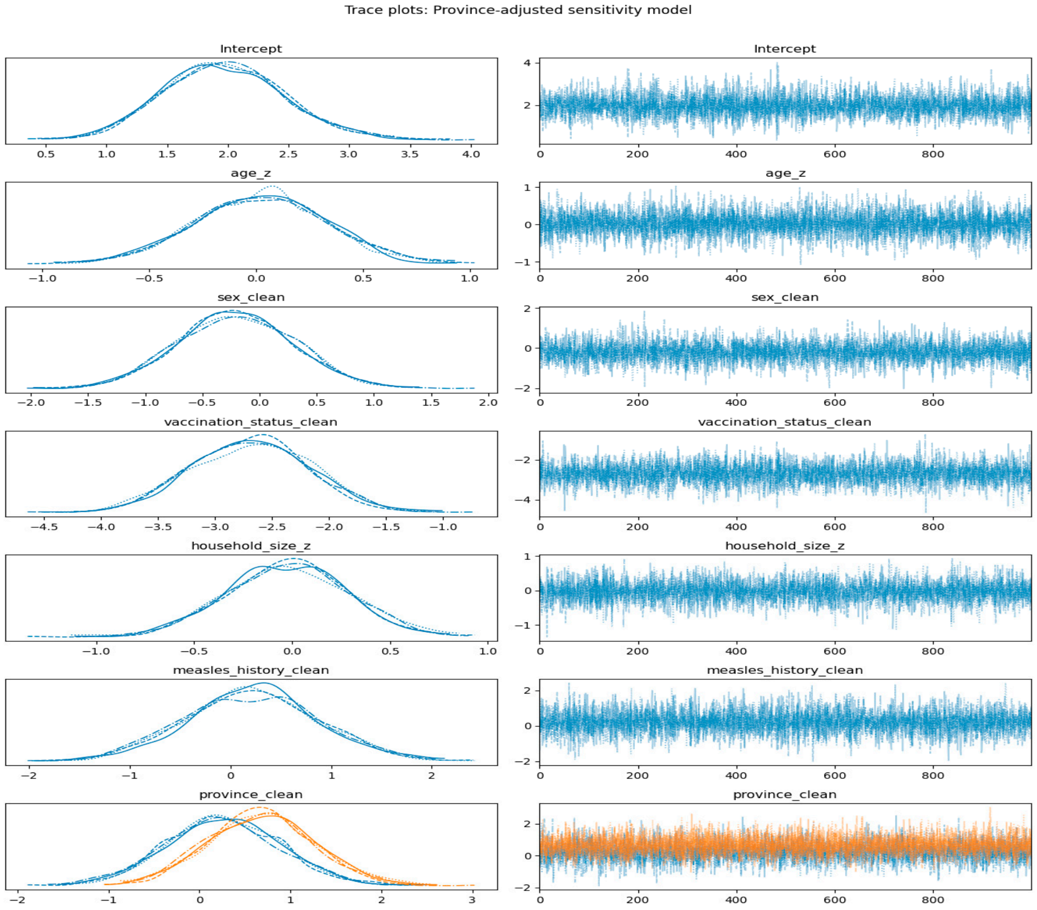

Supplement: Supplementary file 1 [file tropicalmed-11-00248-s001.zip › tropicalmed-4495648-supplementary.pdf]
